# Genetic investigation of XPA gene: high frequency of the c.682C>T mutation in Moroccan XP patients with moderate clinical profile

**DOI:** 10.1186/s13104-017-3042-6

**Published:** 2017-12-06

**Authors:** Zineb Kindil, Mohamed Amine Senhaji, Amina Bakhchane, Hicham Charoute, Soumia Chihab, Sellama Nadifi, Abdelhamid Barakat

**Affiliations:** 1 Human Molecular Genetics Laboratory, Institut Pasteur du Maroc, 1, Place Louis Pasteur, 20360 Casablanca, Morocco; 2 Laboratory of Genetics and Molecular Pathology, Faculty of Medicine, Hassan II University, Casablanca, Morocco; 3 Department of Dermatology, Hospital University Ibn Rochd, Casablanca, Morocco

**Keywords:** Xeroderma pigmentosum, *XPA*, Mutation, Morocco

## Abstract

**Objective:**

Xeroderma pigmentosum (XP) is a genetically and clinically heterogeneous disease, associated with an inherited defect in one of eight different genes (*XPA* to *XPG* and *XPV*). In addition to the early onset of the skin manifestations, the XP group A is marked by the presence of a mild to severe neural disorders which appear tardily and worsens with age. In this study, 9 patients with moderate clinical profile belonging to 6 *XP* families were recruited to determine the *XPA* mutational spectrum in Morocco, using the direct sequencing of the whole coding region of the *XPA* gene.

**Results:**

The genetic investigation of the *XPA* gene showed that 7 from 9 patients were homozygous for the c.682C>T, p.Arg228X mutation, while all their investigated family members were heterozygous. The frequency of this mutation was estimated to be 83.33% (5/6 families) .The molecular analysis of the 5 other exons of the *XPA* gene, showed that the 2 negative siblings carried no mutation in the *XPA* gene. This finding suggests that c.682C>T (p.Arg228X) mutation is relatively associated with moderate phenotype in XP group A Moroccan families; this result will also contribute to improve the molecular diagnosis of XP disease in Moroccan patients.

## Introduction

Xeroderma pigmentosum (XP) is a rare disease inherited under the autosomal recessive mode. It is clinically characterized by sunlight hypersensitivity, pigmentary changes, premature skin ageing, malignant skin and eyes tumor development [1, 2]. This genodermatosis is responsible evenly for a markedly elevated risk of developing other skin and ocular disorders with or without neurological abnormalities [2–5]. Xeroderma pigmentosum disease shows also a high genetic heterogeneity, it has been classified into eight complementation groups (*XPA* to *XPG* and *XPV*) [6]. The *XPC*, *XPD* and *XPA* complementation groups present the most frequent forms of Xeroderma pigmentosum in Europe, North Africa, Japan and the USA, as they are responsible for about 90% of XP patients worldwide [7]. In Japan about 60% of XP patients belong to group A, moreover, nearly 1% of Japanese population shares the (c.390-1G>C) *XPA* gene mutation with a founder effect [8]. The *XPA* human gene ensures a key role in verification of protein damage during the NER, this gene is composed of 6 exons and encodes a 273 amino acid protein which is involved in DNA excision repair pathway [9]. The *XPA* is a highly mutated gene, and about 32 mutations with different degree of severity have been identified [according to the Human Gene Mutation Database (HGMD): http://www.hgmd.cf.ac.uk/ac/index.php] (Table 1). This explains the fact that the XP group A patients present high heterogeneity of cutaneous and ocular symptoms, which are qualified as moderates comparing with the XPC group, who is known as the most severe form of Xeroderma Pigmentosum. Unlike the XP group C phenotype, XP group A patients present also neurological abnormalities that appeared tardily and independently from UV-induced DNA lesions [10, 11].

**Table 1:** Number and different type of mutation in *XPA* human gene (The Human Gene Mutation Database: HGMD)

Mutation type	Number of mutation
Missense/nonsense	12
Splicing	9
Regulatory	1
Small deletions	6
Small insertions	3
Small indels	1
Gross deletions	0
Gross insertions/duplications	0
Complex rearrangements	0
Repeat variations	0
Total of public mutation	32

In Morocco, prevalence of the xeroderma pigmentosum is approximately 1/80 504, which is higher than that found in Europe and the USA [12]. Soufir et al. have reported that *XPC* gene is the major cause of xeroderma pigmentosum in North Africa, this study showed also that the founder mutation 1643-1644delTG in *XPC* gene is responsible for a high proportion of XP cases [13]. This founder mutation was estimated to be responsible of more than 76% XP in Moroccan patients [14]. To the best of our knowledge, there is no study investigating the spectrum of *XPA* gene mutations in the Moroccan population. Thus, 9 Moroccan patients suspected to be XP group A were analyzed; they present different degrees of severity of neuronal disorders and their age range from 7 to 33. This work aims to describe the mutation spectrum of the *XPA* gene in Moroccan XP patients, and clarify its involvement in molecular diagnosis of XP group A Moroccan patients.

## Main text

### Methods

#### Patients

We recruited 9 *XPA* patients (5 male and 4 female individuals), belonging to 6 unrelated families to describe the genetic profile of *XPA* diseases in Moroccan XP patients. The Classification of these patients as XP group A was based on clinical symptoms observed in almost the totality of them, including an abnormal neurological development, which worsens with age and moderate skin lesions which start earlier than neurological signs. One of our patients, a 7 years child (XP43.01) whose recruitment was based in his family history, shows no neurological disorders. Our participants all originated from different regions of Morocco and are all diagnosed and treated at the department of Dermatology in Ibn Rochd University Hospital in Casablanca.

A detailed questionnaire giving all information regarding the clinical data of each patient was filled in. An informed consent was obtained from patients as well as their relatives and was approved by the local committee on research ethics of the Pasteur Institute in Morocco.

#### Molecular analysis

For all patients and their relatives, the DNA extraction from whole blood was carrying out according to phenol chloroform standard protocol [15]. To disclose the presence of genetic variations in our patients, the six exons of *XPA* gene were amplified using specific primers (Table 2). The PCRs were done in particular conditions included initial denaturation at 95 °C for 5 min, 35 cycles of denaturation at 94 °C for 30 s, annealing at a specific temperature which change depending of the primer couple for 35 s, extension at 72 °C for 40 s, and final elongation at 72 °C for 7 min.

**Table 2:** list of primers used in PCR amplification of different exons of the *XPA* gene

Exon	Size (pb)	Forward primer	Reverse primer
1	430	5-AGAGAGCAGGTAGTTAGGCGGG	5-CGGGGAGAGGGAAGGGGAAAG
2	320	5-TTGTGGACATCCTTGTGTTGTTTG	5-TGGCATTATTTAGCATCACTTTGC
3	417	5-GTCAGGCATTGCATACATGCTG	5-GGCATCCTTCCTATTTTATGGGG
4	337	5-GCTGTGTGTGCCCCTAAGTTGC	5-AGCAAAAGCCAAACCAATTATGAC
5	496	5-AGCATACGTTTACTGACAGTTTCATAGG	5-CTTGAAGACCAACATACTGAGGGC
6^a^	594	5_-GTGAGGTAAGAAAGTAAGTTTGCCAAG	5_-TCTAGCACTCAGCTCCCATCTCTG
6^b^	544	5_-GTTTCAGTGAAGGTCACCTGGC	5_-GGTTGGTAAATGCTCAGTAAATGTTAGC

^a^The first fragment of exon 6

^b^The second fragment of exon 6

PCRs were performed in 15 µl final mixture volume containing 30–50 ng of DNA, 6 pmol of each primer, 200 µM of dNTPs, 3 mM of MgCl_2_, 1× PCR buffer, 0.75 U of GoTaq polymerase (Promega, Madison, USA). Purified PCR product was sequenced using the BigDye Terminator v 1.1 Standard Kit according to the manufacturer’s recommendations (Applied Biosystems, Foster City, CA, USA) using ABI 3130 Genetic Analyzer.

### Results

This study involved 6 unrelated XPA Moroccans families, including 9 patients. All patients developed pokilodermia as first symptom of XP at a mean age of 36 month, this pokilodermia was present in all patients’ sun exposed zones (face and hand), and was more marked in patients with low sun protection, 44.44% (4/9) of our patients showed telangiectasia, and only one woman suffered from malignant tumors, she developed an non melanoma skin cancer (NMSC) at 16 years old, the Pathological analysis demonstrated an 5 × 7.5 × 3 mm basal cell carcinoma (BCC) located in the left side of the base of her nose, a later dermatoscopy examination demonstrated that she has also developed two benign tumors at 20 and 23 years old. No ocular malignancies were noticed in our patients. However five patients had photophobia and three showed keratitis in one or both eyes, one patient *XP22.02* had a repetitive eye inflammation which was first diagnosed at the age of 10 years old. All our recruited patients was born with normal size and weight, an neurological abnormalities progression was shown in 8 among them; including a low sensorineural hearing loss observed in two siblings XP16.01 and XP16.02, a 33 years woman XP39.01 showed a severe mental and psychomotor retardation which progress in a loss of ability to walk, speech and motion disorders; according to her family history, two of her sisters had a typical *XPA* clinical profile, they also developed an progressive intellectual impairment and died in their early adulthood. Those patient’s sisters hadn’t been sequenced for the *XPA* gene, but their medical history mentioned that during their lives they developed all clinical signs characteristic of the Xeroderma pigmentosum type A. One young boy XP43.01 had a normal neurological development at the moment of his recruitment (Table 3), his family history indicate that two members of his maternal family (aunt and uncle), had a XPA clinical profile, similarly to the XP39.01 sister’s, they developed a severe neurological troubles in their adulthood, before dying from cancer at the age of 29 and 34 years old respectively. The other patients aged between 12 and 18 years old had no similar antecedents in their families.

**Table 3:** Clinical symptoms of *XPA* patients

Family code	Number of patient/family	Sex	Age	Age of first consultation (m)	Symptoms		
					Dermatologic	Ocular	Neurologic
*XP16*	2	F	31	84	++	+	+
		F	32	72	++	+	+
*XP22*	2	M	15	60	++	−	+
		F	18	18	+	+	++
*XP31*	1	M	12	36	+	−	+
*XP32*	2	M	14	72	+	+	+
		M	16	60	+	+	++
*XP39*	1	F	33	120	+++	+	+++
*XP43*	1	M	7	18	+	−	−

**Fig. 1:**
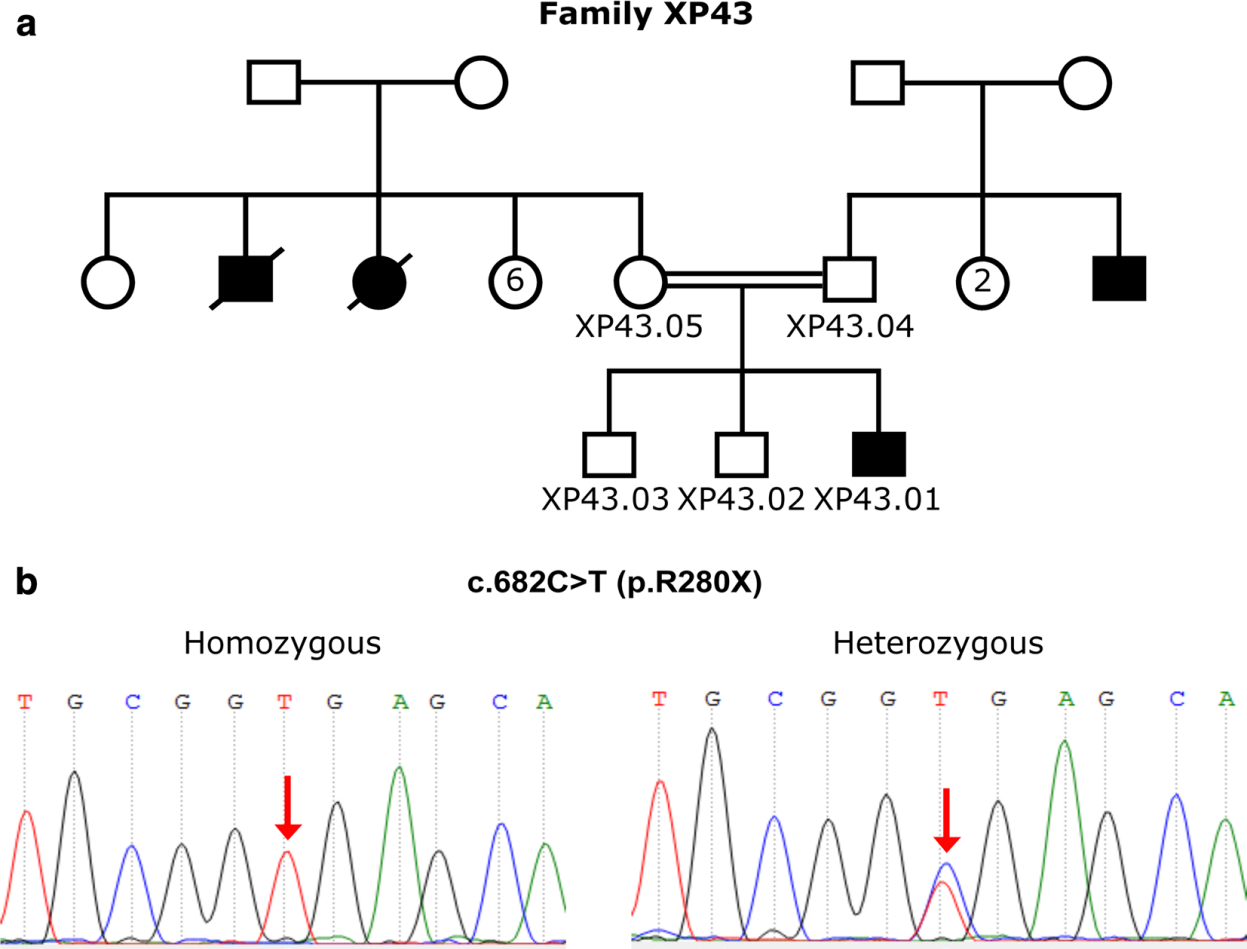
**a** Pedigree of the family *XP43*. **b** Localization of the mutation C.682C>T (P.R228X)

The screening of the coding region of the *XPA* gene disclosed the presence in homozygote state of the recurrent mutation c.682C>T (p.Arg228Ter) in 7/9 XP patients. This punctual variation localized in the exon 6 of the *XPA* gene leads to a premature termination of the encoded protein. Furthermore, all tested parents and healthy relatives of these patients were heterozygous for this mutation (Fig. 1). Additional investigation showed that 2 female siblings had no mutations in the whole *XPA* gene; even so they present a moderate clinical profile with mild neural retardation, this clinical feature is maybe related with molecular variations in other *XP* gene (*XPB*, *XPD* or *XPF*) [16, 17].

### Discussion

Xeroderma pigmentosum is genetically and clinically heterogonous disease, its incidence was estimated at 1/1,000,000 in the United States and Europe, 1/20,000–100,000 in Japan [8, 16], and around 1/10,000–50,000 in North Africa and the Middle East [17, 18]. Eight genes were identified as implicated in this rare genodermatosis among them the *XPA* gene [6], which is characterized by neurological defects [6]. In Morocco, no data concerning the XP group A incidence is available. However in this study a clinical and mutational investigation of *XPA* complementation group is performed in Moroccan patients, in order to contribute to improve diagnosis for XP group A patients and to establish a genetic counseling for their families.

The majority of our patients 88.88% (8/9) were born from consanguineous parents; they are aged between 7 and 33 years with average age of 16.42 years. Moreover, almost all of them showed moderate skin and ocular symptoms. Neurological disorders were also observed especially in aged patients. The patient XP43.01 too young to show a mental abnormal development had a normal neurological profile.

**Fig. 2:**
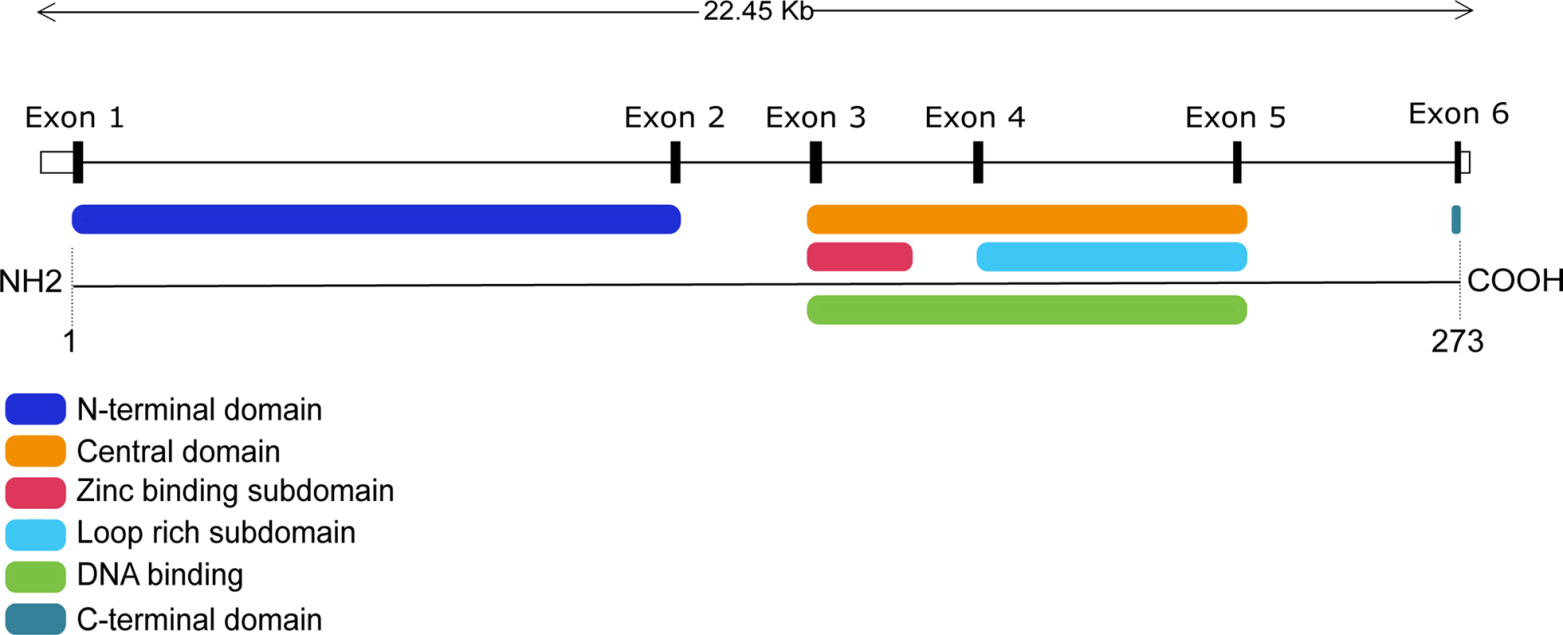
Mapping of *XPA* gene structure showing *XPA* exons and different *XPA* reactive domains (adapted from Ref. [19])

The *XPA* is one of six XP protein factors overriding in human NER machinery [8]. Nowadays, over thirty mutations affecting the *XPA* gene had been listed at the Human Gene Mutation Database; the majority of them are Missense variations (http://www.hgmd.cf.ac.uk/ac/index.php). The direct sequencing of the exon 6 of *XPA* gene showed that about 83% of screened families bear the c.682C>T (p.Arg228X) point mutation, this mutation correspond to C to T transition at position 682 of the coding DNA, and leads to a truncated protein. This nonsense mutation occurs in the C-terminal domain of XPA protein, and this could explain the moderate clinical manifestations of our XP group A patients; since the severity of the clinical phenotype is observed when mutations are located in the DNA binding region (aa 98–219) (Fig. 2). This moderate phenotype was also detected in American, European and Japanese families who are sharing this punctual mutation [19]. In Tunisia, six out of seven XP group A patients had this nonsense mutation with a frequency of 86% [20]. In addition, a previous study has shown that all XP group A Tunisian patients with moderate phenotype had this mutation with founder effect [21]. The same mutation was also identified in Algerian XP group A patients [22]. A study leading by Soufir et al. including 66 unrelated XP families from North Africa, showed that the c.682C>T (p.Arg228X) mutation was present in all XP group A analyzed patients with a frequency of 12% [13]. This suggested that this mutation may have a common founder effect in the North African region.

Severe clinical phenotype is usually observed when patients had mutations within exons 3–5, as reported in four Egyptians [23] and one Iranian patient [24]. A very severe abnormalities were also observed in a Japanese patient who present a mutation located at intron 3 in the splice site [25].

On the other hand, the molecular investigation in patients with no mutation in exon 6, has demonstrated that those two young siblings don’t share any molecular variation in the others *XPA* gene exons. As perspective, those two patients will be analyzed for the *XPB*, *XPD* and *XPF* genes responsible of rare forms of xeroderma pigmentosum with neurological disorders [26, 27].

In conclusion, our finding suggested that the c.682C>T (p.Arg228X) mutation is relatively associated with moderate clinical profile in XP group A Moroccan patients.

## Limitations

This work in which the *XPA* gene were analyzed for the first time in the Moroccan population will provide a basis for the prenatal diagnosis and genetic counseling. However, the sample size is relatively small and further studies are necessary to determine the spectrum of *XPA* gene mutations is Moroccan patients.

## Abbreviations

BCC: basal cell carcinoma
NER: nucleotide excision repair
NMSC: melanoma skin cancer
PCR: polymerase chain reaction
*XP*: xeroderma pigmentosum
*XPA*: xeroderma pigmentosum, complementation group A
*XPC*: xeroderma pigmentosum, complementation group C
*XPD*: xeroderma pigmentosum complementary group D
*XPG*: xeroderma pigmentosum complementary group G
*XPV*: xeroderma pigmentosum, variant type

## References

[1] Butt FMA, Moshi JR, Owibingire S, Chindia ML (2010). Xeroderma pigmentosum: a review and case series. J Cranio-Maxillo-fac Surg Off Publ Eur Assoc Cranio-Maxillo-fac Surg..

[2] Tamura D, DiGiovanna JJ, Kraemer KH (2010). Founder mutations in xeroderma pigmentosum. J Invest Dermatol.

[3] Stary A, Sarasin A (2002). The genetics of the hereditary xeroderma pigmentosum syndrome. Biochimie.

[4] Kraemer KH, Lee MM, Scotto J (1987). Xeroderma pigmentosum. Cutaneous, ocular, and neurologic abnormalities in 830 published cases. Arch Dermatol..

[5] Sugasawa K, Ng JM, Masutani C, Iwai S, van der Spek PJ, Eker AP (1998). Xeroderma pigmentosum group C protein complex is the initiator of global genome nucleotide excision repair. Mol Cell.

[6] Lehmann AR (2003). DNA repair-deficient diseases, xeroderma pigmentosum, Cockayne syndrome and trichothiodystrophy. Biochimie..

[7] Legerski R, Peterson C (1992). Expression cloning of a human DNA repair gene involved in xeroderma pigmentosum group C. Nature.

[8] Hirai Y, Kodama Y, Moriwaki S-I, Noda A, Cullings HM, Macphee DG (2006). Heterozygous individuals bearing a founder mutation in the XPA DNA repair gene comprise nearly 1% of the Japanese population. Mutat Res.

[9] Legerski RJ, Liu P, Li L, Peterson CA, Zhao Y, Leach RJ (1994). Assignment of xeroderma pigmentosum group C (XPC) gene to chromosome 3p25. Genomics.

[10] Laposa RR, Cleaver JE (2001). DNA repair on the brain. Proc Natl Acad Sci USA.

[11] Ben Rekaya M, Messaoud O, Talmoudi F, Nouira S, Ouragini H, Amouri A (2009). High frequency of the V548A fs X572 XPC mutation in Tunisia: implication for molecular diagnosis. J Hum Genet.

[12] Doubaj Y, Laarabi F-Z, Chafai Elalaoui S, Barkat A, Sefiani A (2012). Carrier frequency of the recurrent mutation c.1643_1644delTG in the XPC gene and birth prevalence of the xeroderma pigmentosum in Morocco. J Dermatol.

[13] Soufir N, Ged C, Bourillon A, Austerlitz F, Chemin C, Stary A (2010). A prevalent mutation with founder effect in xeroderma pigmentosum group C from north Africa. J Invest Dermatol.

[14] Senhaji MA, Abidi O, Nadifi S, Benchikhi H, Khadir K, Ben Rekaya M (2013). c.1643_1644delTG XPC mutation is more frequent in Moroccan patients with xeroderma pigmentosum. Arch Dermatol Res.

[15] Grimberg J, Nawoschik S, Belluscio L, McKee R, Turck A, Eisenberg A (1989). A simple and efficient non-organic procedure for the isolation of genomic DNA from blood. Nucleic Acids Res.

[16] Moriwaki S, Kraemer KH (2001). Xeroderma pigmentosum–bridging a gap between clinic and laboratory. Photodermatol Photoimmunol Photomed.

[17] Cartault F, Nava C, Malbrunot A-C, Munier P, Hebert J-C, N’Guyen P (2011). A new XPC gene splicing mutation has lead to the highest worldwide prevalence of xeroderma pigmentosum in black Mahori patients. DNA Repair Amst.

[18] Zghal M, El-Fekih N, Fazaa B, Fredj M, Zhioua R, Mokhtar I (2005). Xeroderma pigmentosum. Cutaneous, ocular, and neurologic abnormalities in 49 Tunisian cases. Tunis Méd.

[19] States J, McDuffie E, Myrand S, McDowell M, Cleaver J (1998). Distribution of mutations in the human xeroderma pigmentosum group A gene and their relationships to the functional regions of the DNA damage recognition protein. Hum Mutat.

[20] Nishigori C, Zghal M, Yagi T, Imamura S, Komoun MR, Takebe H (1993). High prevalence of the point mutation in exon 6 of the xeroderma pigmentosum group A-complementing (XPAC) gene in xeroderma pigmentosum group A patients in Tunisia. Am J Hum Genet.

[21] Messaoud O, Ben Rekaya M, Cherif W, Talmoudi F, Boussen H, Mokhtar I (2010). Genetic homogeneity of mutational spectrum of group-A xeroderma pigmentosum in Tunisian patients. Int J Dermatol.

[22] Bensenouci S, Louhibi L, De Verneuil H, Mahmoudi K, Saidi-Mehtar N. Diagnosis of xeroderma pigmentosum Groups A and C by detection of two prevalent mutations in West Algerian population: a rapid genotyping tool for the frequent XPC mutation c.1643_1644delTG. BioMed Res Int. 2016. Disponible sur: http://www.ncbi.nlm.nih.gov/pmc/articles/PMC4931069/. Accessed 18 Jul 2017. 10.1155/2016/2180946 PMC4931069 27413738

[23] Amr K, Messaoud O, El Darouti M, Abdelhak S, El-Kamah G (2014). Mutational spectrum of xeroderma pigmentosum group A in Egyptian patients. Gene.

[24] Ghafouri-Fard S, Fardaei M, Miryounesi M (2016). A novel 5 nucleotide deletion in XPA gene is associated with severe neurological abnormalities. Gene.

[25] Nishigori C, Moriwaki S, Takebe H, Tanaka T, Imamura S (1994). Gene alterations and clinical characteristics of xeroderma pigmentosum group A patients in Japan. Arch Dermatol.

[26] Dupuy A, Sarasin A (2015). DNA damage and gene therapy of xeroderma pigmentosum, a human DNA repair-deficient disease. Mutat Res.

[27] Moriwaki S, Nishigori C, Imamura S, Yagi T, Takahashi C, Fujimoto N (1993). A case of xeroderma pigmentosum complementation group F with neurological abnormalities. Br J Dermatol.

